# MicroRNA-224 inhibits proliferation and migration of breast cancer cells by down-regulating fizzled 5 expression

**DOI:** 10.18632/oncotarget.9734

**Published:** 2016-05-31

**Authors:** Feng Liu, Yang Liu, Jingling Shen, Guoqiang Zhang, Jiguang Han

**Affiliations:** ^1^ Department of Breast Surgery, Cancer Hospital of Harbin Medical University, Harbin 150081, China; ^2^ Department of Histology and Embryology, Harbin Medical University, Harbin 150081, China

**Keywords:** MiR-224, Wnt/β-catenin signaling, proliferation, migration, breast cancer cells

## Abstract

The Wnt/β-catenin signaling is crucial for the proliferation and migration of breast cancer cells. However, the expression of microRNA-224 (miR-224) in the different types of breast cancers and its role in the Wnt/β-catenin signaling and the proliferation and migration of breast cancer cells are poorly understood. In this study, the levels of miR-224 in different types of breast cancer tissues and cell lines were examined by quantitative RT-PCR and the potential targets of miR-224 in the Wnt/β-catenin signaling were investigated. The effects of altered miR-224 expression on the frequency of CD44^+^CD24^−^ cancer stem-like cells (CSC), proliferation and migration of MCF-7 and MDA-MB-231 cells were examined by flow cytometry, MTT and transwell migration. We found that the levels of miR-224 expression in different types of breast cancer tissues and cell lines were associated inversely with aggressiveness of breast cancers. Enhanced miR-224 expression significantly reduced the fizzled 5-regulated luciferase activity in 293T cells, fizzled 5 expression in MCF-7 and MDA-MB-231 cells, the β-dependent luciferase activity in MCF-7 cells, and the nuclear translocation of β-catenin in MDA-MB-231 cells. miR-224 inhibition significantly increased the percentages of CSC in MCF-7 cells and enhanced proliferation and migration of MCF-7 cells. Enhanced miR-224 expression inhibited proliferation and migration of MDA-MB-231 cells, and the growth of implanted breast cancers *in vivo*. Induction of frizzled 5 over-expression mitigated the miR-224-mediated inhibition of breast cancer cell proliferation. Collectively, these data indicated that miR-224 down-regulated the Wnt/β-catenin signaling possibly by binding to frizzled 5 and inhibited proliferation and migration of breast cancer cells.

## INTRODUCTION

Aberrant activation of the Wnt/β-catenin signaling promotes proliferation and migration of breast cancer cells [1, 2]. The Wnt binds to its co-receptors of Frizzled to activate Dsh, which recruits glycogen synthase kinase-3 beta (GSK-3b), promoting β-catenin nuclear translocation and downstream gene expression [3]. Hence, down-regulating the Wnt/β-catenin signaling should inhibit proliferation and migration of breast cancer cells.

MicroRNA can bind to the 3′ terminal untranslation region (3′UTR) of targeted mRNAs to inhibit their translation and to promote their degradation [4–6]. Previous studies have shown that miR-224 can bind to the tumor suppressors of TNFα-induced protein 1 (TNFAIP1) and Smad4, Raf kinase inhibitor protein (RKIP), apoptosis inhibitor-5 (API-5), PH domain leucine-rich-repeat protein phosphatase 1 (PHLPP1), and PHLPP2 to promote the survival and proliferation of colorectal cancer (CRC) and hepatocellular carcinoma (HCC), but bind to the TRIB1 to promote apoptosis of prostatic cancer cells [7–11]. Furthermore, miR224 binds to the Smad4 and Homeobox D10 (HOXD10) to promote migration of HCC and CRC [8, 12] while it binds to the TPD52 to inhibit migration of prostatic cancer PCa cells [13]. Apparently, the regulatory effects of miR-224 are tissue-specific and depend on the dynamic balance of different signal pathways that regulate proliferation, apoptosis and migration of cancer cells.

There are a few studies on miR-224 regulating proliferation and migration of breast cancer cells. A previous study indicates that high levels of miR-224 are detected in MDA-MB-231 cells to promote migration of MDA-MB-231 cells by targeting the Raf kinase inhibitor protein (RKIP) [14]. However, another study reveals that miR-224 expression is down-regulated during lobular neoplasia progression [15], and miR-224 inhibits migration and invasion of MCF-7 and MDA-MB-231 cells by targeting the CDC42 and CXCR4 [16]. Currently, little is known about the levels of miR-224 expression in different types of breast cancers and about how miR-224 regulates the Wnt/β-catenin signaling, proliferation and migration of breast cancer cells, particularly for breast cancer stem-like cells (CSC).

The goal of current studies is to determine the expression of miR-224 in different types of breast cancers and the role of miR-224 in the Wnt/β-catenin signaling, proliferation and migration of breast cancer cells. We examined the miR-224 expression in different types of breast cancer tissues and breast cancer cell lines as well as the levels of serum miR-224 in patients with different types of breast cancers by quantitative RT-PCR. Furthermore, we characterized the potential targets of miR-224 in the Wnt/β-catenin signaling by bioinformatics analysis, dual luciferase, Western blot and immunofluorescence assays. Subsequently, we determined the effect of altered miR-224 expression on the proliferation, migration and wound healing of breast cancer cells *in vitro* and the growth of implanted xenograft tumors *in vivo*. Our data indicated that miR-224 inhibited the Wnt/β-catenin signaling, proliferation and migration of breast cancer cells by down-regulating fizzled 5 expression.

## RESULTS

### miR-224 expression is associated inversely with aggressiveness of breast cancer

**Figure 1:**
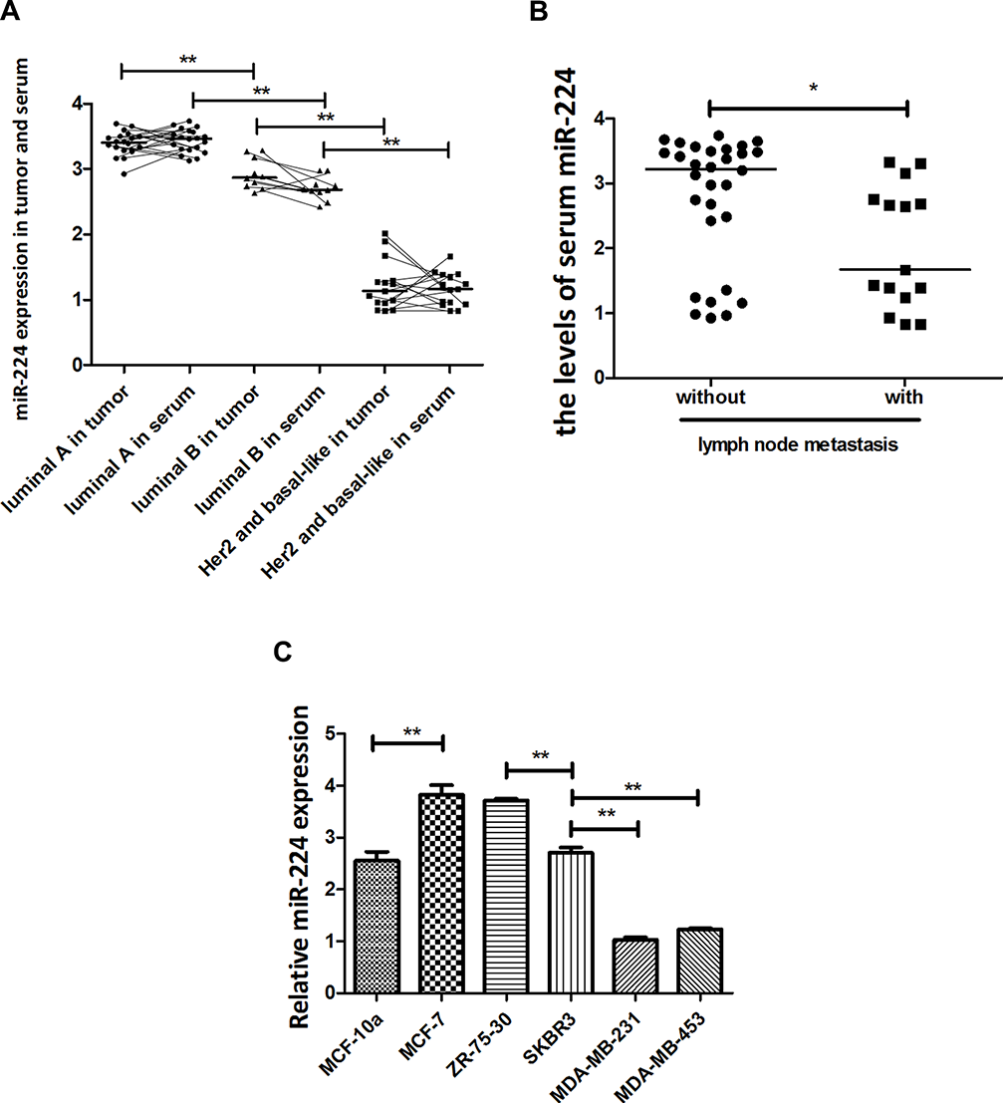
miR-224 expression in breast cancers The relative levels of miR-224 expression in different types of breast cancer tissue and serum samples were determined by quantitative RT-PCR. Furthermore, the relative levels of serum miR-224 in patients (*n* = 15) with or without lymph node metastasis (*n* = 30) were stratified. Finally, the relative levels of miR-224 in different types of breast cancer cell lines were determined. Data are expressed as individual mean values or the mean ± SD of each group of samples from at least 5 separate experiments. (**A**) The levels of miR-224 expression in breast cancer tissues and serum samples. *N* = 20 for luminal A; 10 for luminal B; 15 for Her2^+^/basal-like. (**B**) The levels of serum miR-224 in breast cancer patients with, or without lymph node metastasis. (**C**) The levels of miR-224 expression in different types of breast cancer cell lines. *p < 0.05; **p < 0.01.

miR-224 is an oncogenic regulator in many types of cancers, but the miR-224 expression in the different types of breast cancers has not been clarified. To study the expression and the regulatory function of miR-224, we examined the relative levels of miR-224 expression in 45 different molecular types of breast cancer tissues and their co-responding serum samples by quantitative RT-PCR. The relative levels of miR-224 expression in 10 luminal B tumors were significantly lower than that in the luminal A tumors, but were significantly higher than that in 15 basal-like and Her2^+^ tumors (Figure 1A). A similar pattern of miR-224 was detected in serum samples from these breast cancer patients. Further analysis indicated that the relative levels of serum miR-224 in breast cancer patients with positive lymph node metastasis were significantly lower than that in those without lymph node metastasis (Figure 1B). Moreover, the relative levels of miR-224 in non-tumor MCF-10a were significantly lower than that in luminal MCF-7 and ZR-75-30 cells and were similar to that in Her2^+^ SKBR3 cells (Figure 1C). The relative levels of miR-224 expression in Her2^+^ SKBR3 cells were significantly lower than that in luminal MCF-7 and ZR-75-30 cells, but significantly higher than that in triple negative MDA-MB-231 and MDA-MB-453 cells (Figure 1C). Collectively, mir-224 expression appeared to be inversely associated with aggressiveness of breast cancers in this population.

### Enhanced miR-224 expression reduces Frizzled 5 and inhibits the Wnt/β-catenin signaling in breast cancer cells

**Figure 2:**
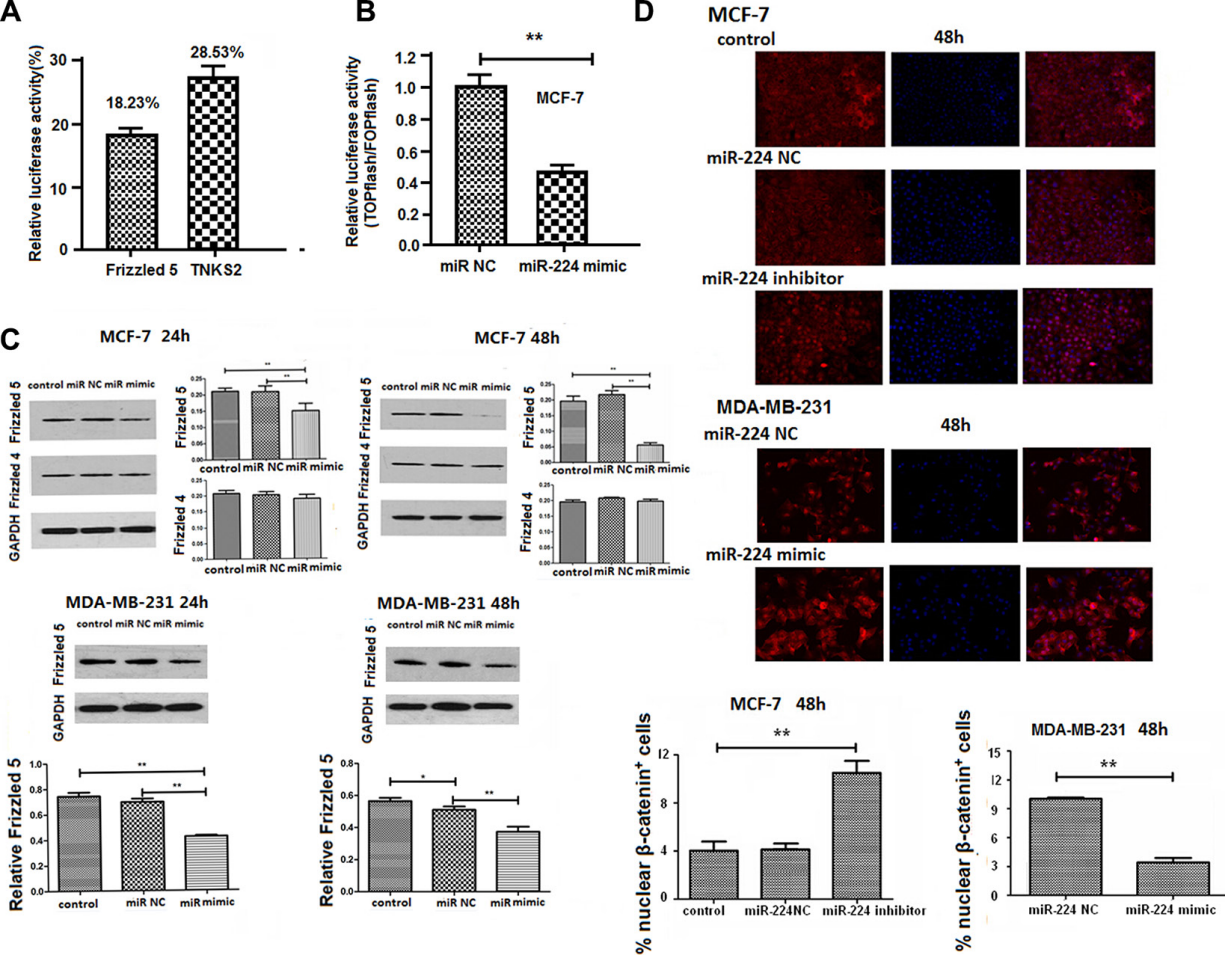
miR-224 reduces Fizzled 5 expression and inhibits the Wnt/β-catenin signaling in breast cancer cells The potential binding sequences of miR-224 in the Wnt/β-catenin signal events were characterized by dual luciferase assays, as described in the Materials and Methods. MCF-7 cells were transfected with miR-224mimic, or miR-224NC for 24 h. The cells in each well were transfected in triplicate with TopFlash plasmid and pRL-TK plasmid for 48 h. The relative levels of β-catenin-dependent firefly luciferase activity in individual samples were determined by the Dual-Luciferase assay. MCF-7 and MDA-MB-231 cells were transfected with, or without, miR-224NC or miR-224mimic for 24 or 48 h. The relative levels of fizzled 4, fizzled 5 and control GAPDH were determined by Western blot. In addition, MCF-7 and MDA-MB-231 cells were transfected with miR-224NC, miR-224inhibitor or miR-224mimic for 48 h and the cytosolic and nuclear β-catenin protein was stained with fluorescent antibodies and DAPI, followed by flow cytometry. Data are representative images and expressed as the mean ± SD of each group of cells from three separate experiments. (**A**) The relative levels of luciferase activity. Transfection with miR-224mimic did not affected the fizzled7 and TNKS2-regulated luciferase activity in 293T cells (data not shown). (**B**) The relative levels of β-catenin dependent luciferase activity. (**C**) Western blot analysis of the fizzled 4 and frizzled 5 expression. (**D**) Immunofluorescent staining of β-catenin^+^ breast cancer cells. *p < 0.05; **p < 0.01.

Aberrant activation of the Wnt/β-catenin signaling promotes proliferation and migration of breast cancer cells (6, 7). We researched the potential sequences in the 3UTR of β-catenin signal events and found that the 3′UTR of Frizzled 4 and Frizzled 5 contained the complementary sequences of miR-224 (Table S1). Accordingly, we tested whether enhanced miR-224 expression could change the 3′UTR of Frizzled 4, Frizzled 5, Frizzled 7, and TNKS2-regulated luciferase activity by dual luciferase assay. We found that transfection of 294T cells with miR-224mimic did not change the Frizzled 7 or TNKS2-regulated luciferase activity (data not shown). In contrast, transfection of 293T cells with miR-224mimic, but not with miR-224NC, significantly reduced the Frizzled 5 and Frizzled 4-regulated luciferase activity by 81.77% and 71.47%, respectively (Figure 2A). Furthermore, transfection with miR-224mimic, but not miR-224NC, significantly reduced the β-catenin-dependent firefly luciferase activity in MCF-7 cells, suggesting that enhanced miR-224 expression inhibited the Wnt/β-catenin signaling (Figure 2B). In addition, transfection with miR-224mimic, but not miR-224NC, significantly reduced the relative levels of Frizzled 5, but not Frizzled 4, expression in MCF-7 and MDA-MB-231 cells (Figure 2C). Moreover, transfection with miR-224inhibitor significantly increased the ratios of nuclear to cytosolic β-catenin^+^ MCF-7 cells (Figure 2D). In contrast, transfection with miR-224mimic significantly decreased the ratios of nuclear to cytosolic β-catenin^+^ MDA-MB-231 cells. Collectively, these data indicated that miR-224 inhibited the Wnt/β-catenin signaling by down-regulating the Frizzled 5 expression in breast cancer cells.

### Reduced miR-224 expression increases the percentages of CD44^+^CD24^−^ CSCs in MCF-7 cells

**Figure 3:**
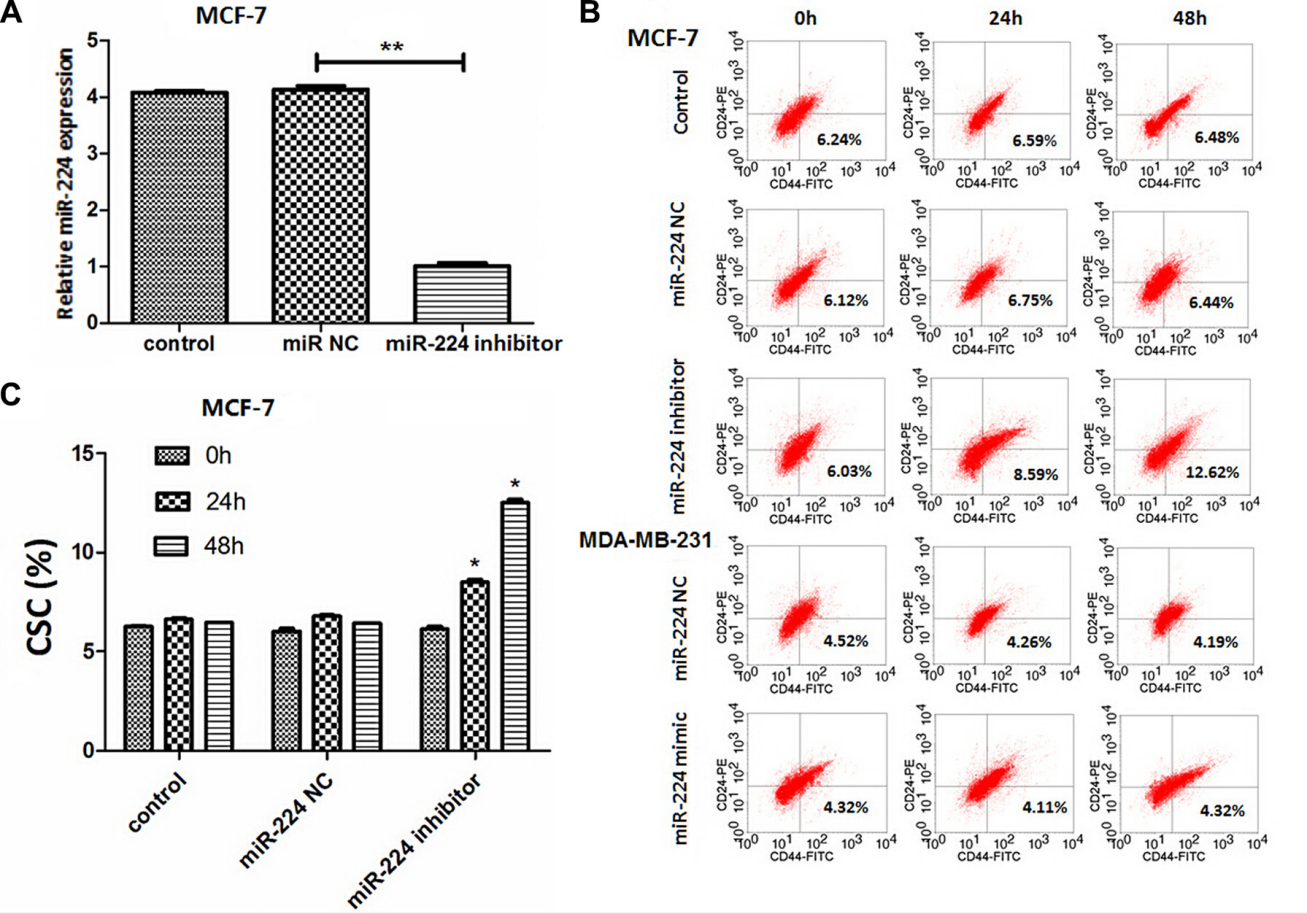
miR-224 inhibition increases the percentages of CD44^+^CD24^−^ CSCs in MCF-7 cells MCF-7 and MDA-MB-231 cells were transfected with, or without, miR-224NC, miR-224inhibitor or miR-224mimic for 24 or 48 h, respectively. The relative levels of miR-224 expression in different groups of cells were determined by quantitative RT-PCR. The cells were stained with FITC-anti-CD44 and PE-anti-CD24 and the frequency of CD44^+^CD24^−^ CSCs was determined by flow cytometry. Data are representative FACS charts and expressed as the mean ± SD of each group of cells from three separate experiments. (**A**) The relative levels of miR-224 expression. Transfection with miR-224mimic increased the levels of miR-224 expression in MDA-MB-231 cells (data not shown). (**B**) Flow cytometry analysis of breast CSCs. (**C**) Quantitative analysis of breast CSCs. Enhanced miR-224 expression did not significantly alter the frequency of CD44^+^CD24^−^ CSCs in MDA-MB-231 cells. *p < 0.05; **p < 0.01.

Breast CSCs are crucial for the recurrence and metastasis of breast cancer. To determine the regulatory function of miR-224, MCF-7 and MDA-MB-231 cells were transfected with, or without, miR-224NC, miR-224 inhibitor or miR-224mimic for 24 or 48 h, respectively. The relative levels of miR-224 in the different groups of cells were determined by quantitative RT-PCR. We found that transfection with miR-224inhibitor, but not with miR-224NC, significantly reduced the levels of miR-224 expression in MCF-7 cells, as compared with that in the untransfected controls (Figure 3A). Transfection with miR-224 mimic, but not miR-224NC, significantly increased the relative levels of miR-224 expression in MDA-MB-231 cells (data not shown). After staining with FITC-anti-CD44 and PE-anti-CD24, we found that transfection with miR-224inhibitor, but not with miR-224NC, significantly increased the percentages of CD44^+^CD24^−^ CSC in MCF-7 cells and its promoting effects tended to be time-dependent (Figure 3B and 3C). However, transfection with miR-224mimic did not significantly change the frequency of CD44^+^CD24^−^ CSC in MDA-MB-231 cells. Hence, miR-224 may inhibit the spontaneous proliferation of breast CSC-like MCF-7 cells *in vitro*.

### miR-224 inhibits proliferation of breast cancer cells

**Figure 4:**
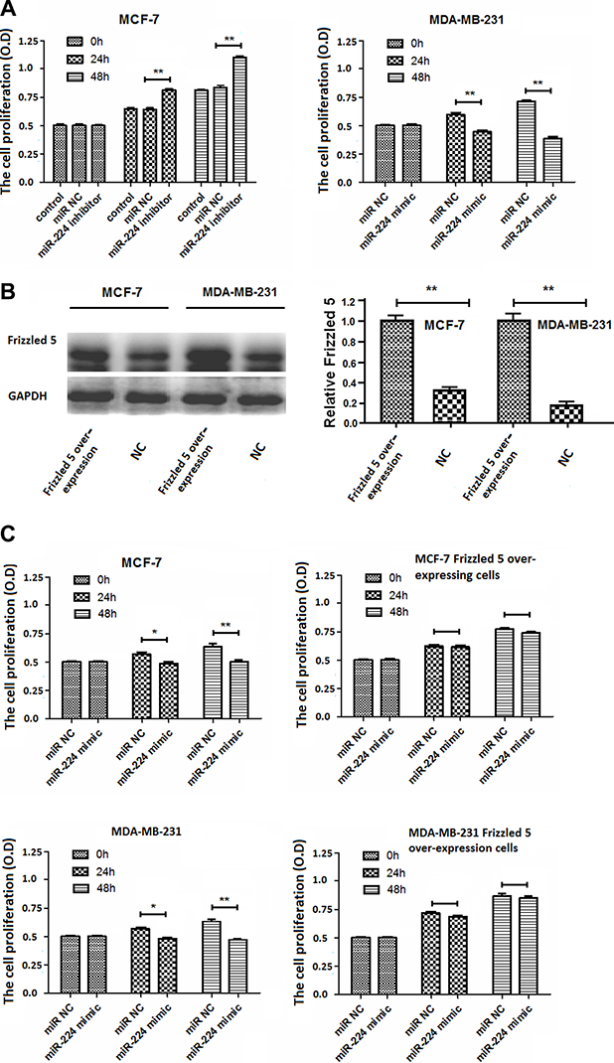
miR-224 inhibits proliferation of breast cancer cells MCF-7 and MDA-MB-231 cells were transfected with, or without, miR-224NC, miR-224inhibitor, or miR-224mimic for 24 or 48 h, respectively. Furthermore, MCF-7 and MDA-MB-231 cells were transfected with a plasmid for frizzled 5 expression, which was characterized by Western blot assays. Subsequently, the cells were transfected with miR-224NC, or miR-224mimic for 48 h. The proliferation of different groups of breast cancer cells was determined by MTT assay. Data are representative images or expressed as the mean ± SD of each group of cells from three separate experiments. (**A**) The proliferation of MCF-7 and MDA-MB-231 cells. (**B**) Western blot analysis of the relative levels of Frizzled 5 expression in MCF-7 and MDA-MB-2231 cells. The relative levels of frizzled 5 in over-expressing MCF-7 and MDA-MB-231 cells were designated as 1. (**C**) The proliferation of Frizzled 5-over-expressing MCF-7 and MDA-MB-231 cells. ^*^p < 0.05; ^**^p < 0.01.

Next, we further examined whether altered miR-224 expression could change proliferation of breast cancer cells by MTT assay. MCF-7 and MDA-MB-231 cells were transfected with, or without, miR-224NC, miR-224inhibitor or miR-224mimic for 24 or 48 h, respectively. We found that transfection with miR-224NC did not change proliferation of MCF-7 and MDA-MB-231 cells (Figure 4). Transfection with miR-224inhibitor significantly enhanced proliferation of MCF-7 cells while transfection with miR-224mimic significantly inhibited proliferation of MDA-MB-231 cells (Figure 4A). Their regulatory effects tended to be time-dependent.

Given that enhanced miR-224 expression significantly reduced the relative levels of Frizzled 5 expression and the Wnt/β-catenin signaling in breast cancer cells, we examined whether induction of Frizzled 5 over-expression could mitigate the miR-224-mediated inhibition of breast cancer cell proliferation. After induction of Frizzled 5 over-expression in MCF-7 and MDA-MB-231 cells (Figure 4B), we found that transfection with miR-224mimic significantly inhibited proliferation of control MCF-7 and MDA-MB-231 cells, but not Frizzled 5-over-expressing MCF-7 and MDA-MB-231 cells (Figure 4C). Therefore, enhanced miR-224 expression inhibited spontaneous proliferation of breast cancer cells *in vitro,* which was abrogated by Frizzled 5 over-expression.

### miR-224 inhibits migration of breast cancer cells

**Figure 5:**
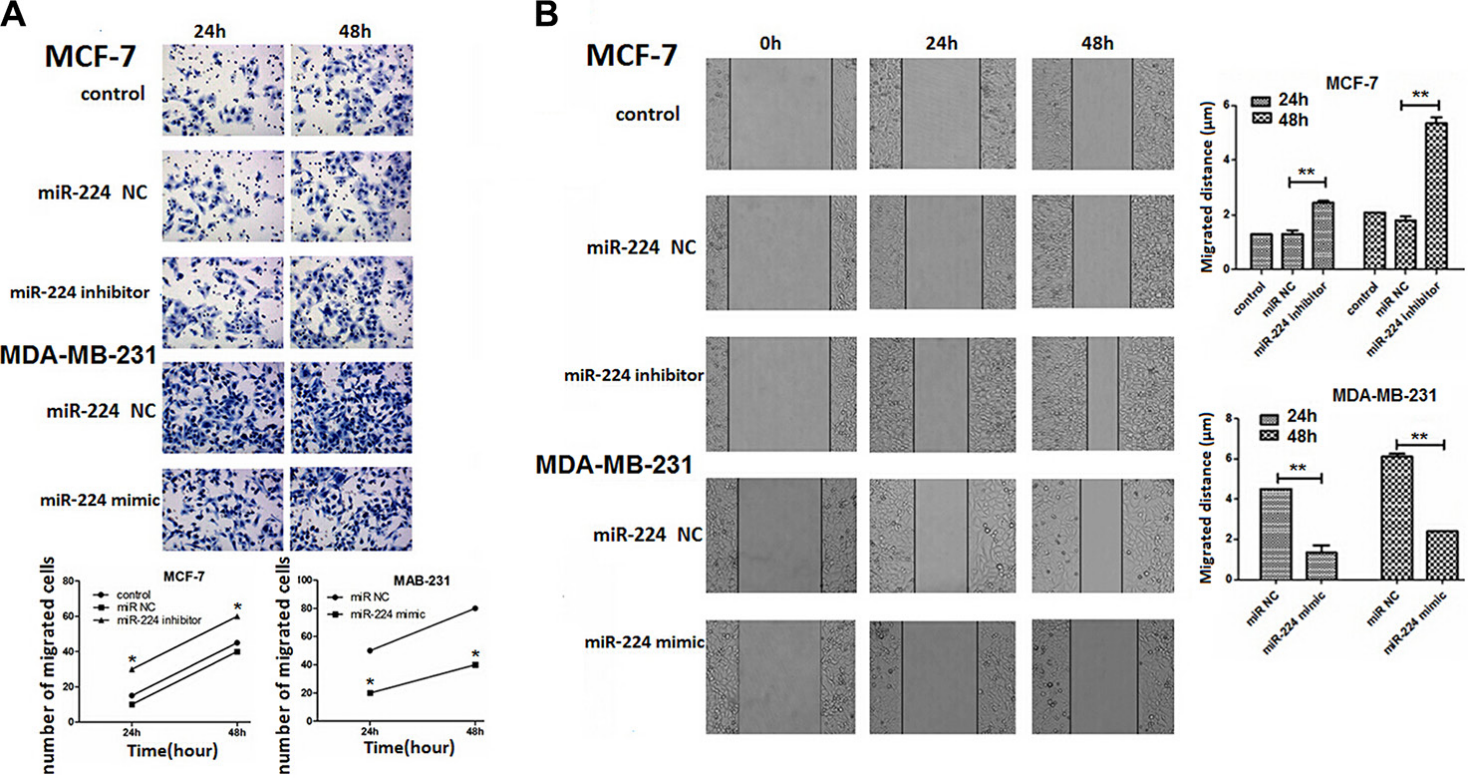
miR-224 inhibits the migration and wound healing of breast cancer cells MCF-7 and MDA-MB-231 cells were transfected with, or without, miR-224NC, miR-224inhibitor, or miR-224mimic, respectively. Four hours after transfection, the migration of different groups of breast cancer cells for the indicated time points was determined by transwell migration assay. In addition, two days after transfection, the wound healing of different groups of cells within 24 or 48 h was tested. Data are representative images and expressed as the mean ± SD of each group of cells from three separate experiments. (**A**) The migration of breast cancer cells. (**B**) The wound healing of breast cancer. *p < 0.05; **p < 0.01.

Next, we examined whether altered miR-224 expression could change the migration of breast cancer cells by transwell migration and wound healing assays. MCF-7 and MDA-MB-231 cells were transfected with miR-224NC, miR-224inhibitor or miR-224mimic for 24 or 48 h. We found that transfection with miR-224NC did not alter the migration of MCF-7 and MDA-MB-231 cells (Figure 5A). While transfection with miR-224inhibitor increased the numbers of migrated MCF-7 cells transfection with miR-224mimic significantly reduced the numbers of migrated MDA-MB-231 cells (Figure 5A). Similarly, transfection with miR-224inhibitor significantly increased the migration distance of MCF-7 cells, but transfection with miR-224mimic significantly decreased the migration distance of MDA-MB-231 cells (Figure 5B). Therefore, miR-224 inhibited the migration of breast cancer cells *in vitro*.

### Enhanced miR-224 expression inhibits the growth of implanted breast cancers *in vivo*

**Figure 6:**
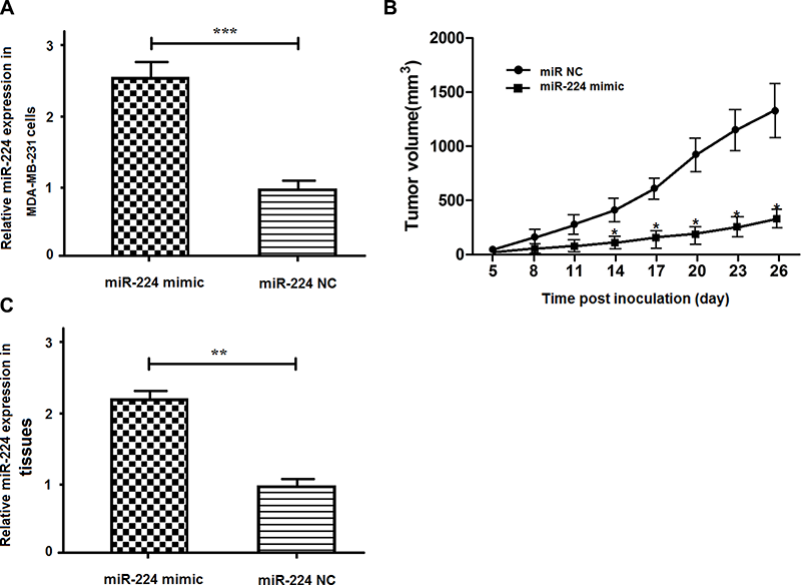
miR-224 inhibits the growth of implanted breast tumors *in vivo* MDA-MB-231 cells were induced for stable expression of miR-224mimic or control miR-224NC and the relative levels of miR-224 expression in the miR-224 stably over-expressing MDA-231/miR-224 mimic or control cells were determined by quantitative RT-PCR. Subsequently, individual groups of cells were implanted into female nude mice and the growth of implanted tumors were monitored at the indicated time points post inoculation. At the end of this experiment (on day 26 post inoculation), the tumors were dissected and the relative levels of miR-224 expression in the different groups of tumors were determined by quantitative RT-PCR. Data are expressed as the mean ± SD of each group of mice (*n* = 8 per group). (**A**) The relative levels of miR-224 expression. (**B**) The tumor volumes. (**C**) The relative levels of miR-224. The relative levels of miR-224 in the miR-2224NC-transfected controls were designated as 1. ^*^p < 0.05; ^***^p < 0.001 vs. the tumors induced by the miR-224 NC-transfected control MDA-MB-231 cells.

Finally, we examined whether enhanced miR-224 could change the dynamic growth of implanted breast cancers *in vivo*. We found that the relative levels of miR-224 in the miR-224mimic stably expressing MDA-MB-231 cells were about 2.5-fold higher than that in the miR-224NC stably expressing MDA-MB-231 cells (Figure 6A). The tumor volumes in the mice implanted with miR-224mimic stably expressing MDA-MB-231 cells were significantly smaller than that implanted with miR-224NC stably expressing MDA-MB-231 cells (Figure 6B). Further analysis indicated the relative levels of miR-224 expression in the tumors induced by miR-224mimic stably expressing MDA-MB-231 cells were significantly higher than that in the tumoes induced by miR-224NC stably expressing cells (Figure 6C). Therefore, enhanced miR-224 expression significantly inhibited the growth of implanted breast cancers in mice.

## DISCUSSION

In this study, we first explored the relative levels of miR-224 in different types of breast cancer tissues by quantitative RT-PCR. We found that the relative levels of miR-224 in luminal B type of breast cancer tissues were significantly lower than that in the luminal A type of tissues, but significantly higher than that in the Her2^+^ and basal-like triple negative breast cancer tissues in this population. Furthermore, we detected a similar pattern of serum miR-224 in these patients. In addition, the relative levels of serum miR-224 in breast cancer patients with lymph node metastasis were significantly lower than that in those without lymph node metastasis. Our findings extended previous observations of down-regulated miR-224 expression in breast lobular neoplasia [15]. To the best of our knowledge, this was the first report that the relative levels of miR-224 in different types of breast cancer tissues were associated inversely with the aggressiveness of breast cancers. Although miR-224 has been shown to promote tumorigenesis of HCC, CRC and gastric cancers [7–9] our data suggest that miR-224 may inhibit the development and progression of breast cancers. Hence, our findings argue that miR-224 may have varying functions in regulating the development and progression of different types of cancers. We are interested in further investigating the potential prognostic values of miR-224 levels in those breast cancer patients.

Aberrant activation of the Wnt/β-catenin signaling is crucial for the proliferation and migration of breast cancer cells. In this study, we found that the 3′UTR of Fizzled 4 and Fizzled 5 contained complementary sequences for miR-224 binding and transfection with miR-224mimic dramatically reduced the luciferase activity regulated by the 3′UTR of Fizzled5 and Frizzled 4 in 293T cells. Furthermore, enhanced miR-224 expression significantly reduced the β-catenin-dependent luciferase activity and the Fizzled 5 expression in MCF-7 and MDA-MB-231 cells, and attenuated β-catenin nuclear translocation in MDA-MB-231 cells. In contrast, knockdown of miR-224 expression inhibited β-catenin nuclear translocation in MCF-7 cells. More interestingly, induction of Frizzled 5 over-expression mitigated the miR-224mimic-mediated inhibition on proliferation of MCF-7 and MDA-MB-231 cells. Collectively, these novel data suggest that miR-224 may bind to the Wnt receptor of Fizzled5 to impair the Wnt/β-catenin signaling in breast cancer cells. Hence, miR-224 may be a natural inhibitor of the Wnt/β-catenin signaling and valuable for intervention of breast cancer. There are several miRNAs that inhibit or enhance the Wnt/β-catenin signaling during the development of different types of tumor, including breast cancer [17]. miR-135 and miR-142 inhibit the adenomatous polyposis coli (APC) expression to enhance the Wnt/β-catenin signaling and tumor cell proliferation and migration [18, 19] while miR-200a, miR-203, miR-214, miR-1826, miR-320 and others attenuate the Wnt/β-catenin signaling by targeting β-catenin to inhibit tumor cell proliferation and migration [20–26]. Hence, these miRNAs form a complex network to regulate the Wnt/β-catenin signaling during the development and progression of tumors.

Inhibition of the Wnt/β-catenin signaling can attenuate proliferation and migration of cancer cells, particularly for CSC [27]. We found that knockdown of miR-224 significantly increased the percentages of CD44^+^CD24^−^ CSCs in MCF-7 cells and proliferation of MCF-7 cells. In contrast, enhanced miR-224 expression attenuated proliferation of MDA-MB-231 cells and growth of implanted breast cancers *in vivo*, but did not significantly change the frequency of CD44^+^CD24^−^ CSCs in MDA-MB-231 cells. Given that the relative levels of miR-224 in MCF-7 cells were significantly higher than that in MDA-MB-231 cells the different responses of CSC to altered levels of miR-224 may be because MCF-7 CSC might be more sensitive to miR-224 regulation than MDA-MB-231 CSC. While miR-224 inhibition promoted migration of MCF-7 cells enhanced miR-224 expression attenuated migration of MDA-MB-231 cells. It is possible that miR-224 reduced Frizzled 5 expression, which impaired the Wnt/β-catenin signaling, the Wnt-mediated β-catenin-independent Planar cell polarity and the Wnt/Ca^++^ signaling to inhibit migration of breast cancer cells [28, 29]. Our data were consistent with a previous report that miR-224 inhibits invasion of MDA-MB-231 by targeting the CDC42 and CXCR4 expression [16]. However, our data were inconsistent with another report that miR-224 binds to the RKIP to promote migration of MDA-MB-231 cells [14]. The distinct outcomes may stem from varying experimental conditions because miR-224 can bind to many molecules, including the RKIP, Smad4, API-5, PHLPP1, PHLPP2, TRIB1, HOXD10, TPD52, CDC42 and CXCR4 [7–9, 14–16]. Hence, the outcome of miR-224-regulated breast cancer cells may depend on the dynamic balance of different signal pathways that regulate their proliferation, apoptosis and migration of cancer cells. We are interested in further investigating the role of miR-224 in regulating the progression of breast cancer *in vivo*.

In summary, our data indicated that the relative levels of miR-224 expression were associated inversely with aggressiveness of different types of breast cancers. Enhanced miR-224 expression reduced the Fizzled 5 expression and inhibited the Wnt/β-catenin signaling, proliferation and migration of breast cancer cells as well as growth of implanted breast cancers in mice. Our data may provide new insights into the proliferation and migration of breast cancer cells. Potentially, our novel findings may aid in design of new therapies for intervention of breast cancer.

## MATERIALS AND METHODS

### Subjects

A total of 45 patients with breast cancer were recruited at the inpatient service of the Harbin Medical University and Dalian Medical University from January 2010 and December 2014. Individual patients with breast cancer were diagnosed by pathological examination of two independent pathologists. Their blood samples were obtained for preparation of serum samples. Their surgical tumor samples were used for molecular classification, and small RNA extraction for characterization of miR-224 expression. The demographic and clinical data of individual patients were obtained from medical records (Table 1). Written informed consent was obtained from individual patients, and the experimental protocol was approved by the Ethics Committee of Harbin Medical University.

**Table 1:** The characteristics of 45 enrolled cases

Various		*n*
Age	≤ 40 y	10
	> 40 y	35
T stage	T1	20
	T2	25
N stage	N0	30
	N1	15
Clinical stge	DCIS	15
	IDC	30
Pathological stage	I	5
	II	30
	III	10
Molecular type	Luminal A	20
	Luminal B	10
	Her2+/basal-like	15

### Cell lines

293T cells, human non-tumor breast epithelial MCF-10a cells and breast carcinoma cell lines, MCF-7 (lumina A, ER^+^, PR^+^, Her2^−^), MDA-MB-231, MDA-MB-453 (basal-like, ER^−^, PR^−^, Her2^−^), ZR-75-30 (luminal ER^+^, Her2^+^) and SKBR3 (ER-, PR-, Her2^+^) were obtained from the American Type Culture Collection (ATCC, Manassas, USA) and cultured in 10% fetal bovine serum (FBS) DMEM medium (complete medium, Invitrogen, Grand island, USA) at 37°C in an atmosphere of 5% CO_2_.

### Quantitative RT-PCR analysis of miR-224

The obvious tumor tissues in individual surgical tumor samples were dissected out. Small RNA was extracted from individual tumor specimens, MCF10a, MCF-7, ZR-75-30, SKBR3, MDA-MB-453 and MDA-MB-231 cells using the miRNeasy Mini Kit (#217004, Qiagen, Valencia, USA). The relative levels of miR-224 were determined by quantitative RT-PCR [2]. Briefly, individual RNA samples were digested with DNase I to remove the contaminated DNA, and the quality of RNA samples were determined. Subsequently, the RNA samples were reversely transcribed into cDNA and the relative levels of miR-224 to the control U6 RNA were determined by quantitative RT-PCR using the All-in one TM miRNA qRT-PCR reagent kit (#R0101L, GeneCopoeia, Rockville, USA). The data were analyzed by 2^−ΔΔCt^. Furthermore, the relative levels of miR-224 in individual serum samples were determined by quantitative RT-PCR.

### Flow cytometry analysis

MCF-7 and MDA-MB-231 cells (1 × 10^5^ cells/well) were cultured in complete medium in 24-well plates overnight and transfected in duplicate with, or without, 2 μM miR-224 NC (UUCUCCGAACGUGUCACGUT TACGUGACACGUUCGGAGAATT), miR-224inhibitor (5′-CAAGUCACUAGUGGUUCCGUU-3′) or miR-224mimic (5-AACGGAACCACUAGUGACUUG-3, Genechem, Shanghai, China) using lipofectamine (Life Technology) for 24 or 48 h. Some cells were used for quantitative analysis of the relative levels of miR-224 expression by quantitative RT-PCR. The remaining cells were stained with FITC-anti-CD44 and PE-anti-CD24 and the percentages of CD44^+^CD24^−^ CSC cells in individual groups of cells were determined by flow cytometry.

### Dual luciferase report assay

The complementary 3′UTR sequences of the canonical Wnt/β-catenin pathway-related mRNAs to which miR-224 potentially bound were searched from RefSeq. We found that miR-224 potentially bound to the 3′UTR of Frizzled 4 and Frizzled 5 (Table S1). Accordingly, the DNA fragments for the 3′UTR of Frizzled 4, Frizzled 5, control Frizzled 7, and TNKS2 were amplified from a cDNA library of breast cancer cells and cloned into the *Xho I* and *Sal I* sites near 3′ of the firefly luciferase gene in the dual-luciferase miRNA target expression vector, pmirGLO, respectively. The generated plasmids were sequenced.

293T cells were cultured in 96-well plates overnight and transfected in triplicate with 0.5 μg individual plasmids that contained the 3′UTR of Frizzled 4, Frizzled 5, Frizzled 7, or TNKS2, together with either 0.75 μM miR-224NC or miR-224 mimic for 48 h. The levels of dual-luciferase activity were measured using the dual-luciferase assay kit (DLR^™^, Promega, Madison, USA), according to the manufacturers' instruction [4].

MCF-7 cells (2 × 10^4^ cells/well) were cultured in complete medium in 96-well plates overnight and transfected in triplicate with 0.75 μM miR-224mimic, or miR-224NC using lipofectamine (Life Technology) for 24 h. The cells in each well were transfected in triplicate with TopFlash (20 ng) plasmid (#12456, Addgene, Cambridge, USA), Renilla luciferase thymidine kinase pRL-TK plasmid (5 ng, E2241, Promega) using lipofectamine for 48 h. The relative levels of β-catenin-dependent firefly luciferase activity in individual samples were determined by the Dual-Luciferase assay. Total value of reporter activity in each sample was normalized to Renilla luciferase activity.

### Western blot assay

MCF-7 and MDA-MB-231 cells were transfected with, or without, miR-224NC or miR-224mimic for 24 or 48 h. The relative levels of Frizzled 4 and Frizzled 5 as well as control GAPDH were determined by Western blot assays [30]. The primary antibodies included goat anti-Frizzled 5 (sc-23223), anti-Frizzled 4 (SC-66451), and rabbit anti-GAPDH (sc-25778, Santa Cruz Biotechnology, Santa Cruz, USA) and negative controls of goat IgG. The relative levels of each interesting protein to GAPDH were determined using the Gel pro4.0.

### Immunofluorescence

MCF-7 and MDA-MB-231 cells were transfected with, or without, miR-224NC, miR-224mimic or miR-224inhibitor for 24 or 48 h. The cells were fixed, permeabilized and stained with PE-anti-β-catenin, followed by counterstaining with DAPI. The immunofluorescent signals were imaged under a fluorescent microscope. The ratios of nuclear and cytoplasmic β-catenin^+^ cells were calculated.

### Proliferation assay

MCF-7 and MDA-MB-231 cells were transfected with, or without, miR-224NC, miR-224mimic or miR-224inhibitor for 24 or 48 h. The proliferation of different groups of cells was determined by MTT. During the last 4-h incubation, the cells were treated with MTT. The resulting formazan was dissolved in DMSO and measured for the absorbance at 540 nm. The data are present as the OD values.

To determine the effect of Fizzled 5 over-expression on miR-224-mediated inhibition of breast cancer cell proliferation, MCF-7 and MDA-MB-231 cells were transfected with recombinant plasmid of pcDNA3.1 as the control or pcDNA-FZD5 (a gift from Prof. Caigang Liu, Dalian Medical University, Dalian, China) for 48 h. The relative levels of Frizzled 5 expression were determined by Western blot using anti-Frizzled 5 (ab75234, Abcom, Cambridge, USA). Subsequently, the cells (2 × 10^4^/well) were transfected in triplicate with miR-224NC or miR-224mimic for 48 h. The proliferation of transfected MCF-7 and MDA-MB-231 cells was determined by MTT.

### Wound healing assay

The effect of miR-224 on the migration of MCF-7 and MDA-MB-231 cells was determined by wound healing assay [31]. Briefly, MCF-7 and MDA-MB-231 cells were transfected with, or without, miR-224NC, miR-224mimic or miR-224inhibitor for 48 h. Individual groups of cells (5 × 10^5^ cells/well) were cultured in triplicate in 24-well plates. When they reached nearly 90% confluence, the cells were scratched with plastic pipette tip across the wells. The cells were continually cultured for 24 or 48 h and imaged. The migration distance of cells was measured using ImageJ software (NIH, Rockville, USA).

### Transwell migration assay

The effect of miR-224 on the migration of MCF-7 and MDA-MB-231 cells was also determined by transwell migration assay [30, 32]. Briefly, MCF-7 and MDA-MB-231 cells were transfected with, or without, miR-224NC, miR-224mimic or miR-224inhibitor for 48 h. Individual groups of cells (1 × 10^5^ cells/well) were cultured in triplicate in the top chambers of 24-well transwell plates (8.0 mm-pore, Corning) and complete medium was added into the bottom chambers. After 24- or 48-h culture, the cells on the surface of the top chamber membrane were removed with a cotton swab. The migrated cells on the bottom surface of the top chamber membranes were fixed with 95% alcohol and stained with hematoxylin solution. The cells were counted in 10 random fields under a light microscope (magnification × 200).

### Xenograft breast tumor model

The experimental protocol was approved by the Animal Research and Care Committee of Harbin Medial University. MDA-MB-231 cells were transfected with pGPU6/GFP/miR-224NC or pGPU6/GFP/miR-224mimic (GenePharma, Shanghai, China). Two days later, the GFP^+^ cells were sorted by flow cytometry and the cells were treated with G418 (Sigma) for three weeks to generate miR-224 and control NC stably high expressing MDA-MB-231 cells. The relative levels of miR-224 expression in different groups of cells were determined by quantitative RT-PCR.

Female athymic nude mice at 6–8 weeks of age were from Silaike Laboratory Animal (Shanghai, China) and housed in a specific pathogen-free facility with free access to autoclaved water and food. Individual mice were randomized and injected subcutaneously with 5 × 10^5^ MDA-231 control, or MDA-231/miR-224 mimic cells (0.1 ml in FBS-free medium) into their fat pad (*n* = 8 per group). The growth of implanted tumors was monitored using a vernier caliper every three days. The volumes of tumors were calculated. At the end of this experiment, the relative levels of miR-224 expression in individual groups of tumors were tested by quantitative RT-PCR.

### Statistical analysis

Data are expressed as the means ± SD or median. The difference among groups was determined by ANOVA and post hoc Fisher's least significant difference. The difference between two groups was analyzed Student *T* test or Mann–Whitney U test using SPSS software, version 16.0. A *P*-value of < 0.05 was considered statistically significant.

## SUPPLEMENTARY MATERIALS TABLE


