# Supplementary material for: MicroRNA-224 inhibits proliferation and migration of breast cancer cells by down-regulating fizzled 5 expression
Source: Oncotarget. 2016 May 31;7(31):49130–42. doi: 10.18632/oncotarget.9734 (PMC5226496; doi:10.18632/oncotarget.9734)
Supplement: Supplementary file 1 [file oncotarget-07-49130-s001.pdf]

MicroRNA-224 inhibits proliferation and migration of breast cancer cells by down-regulating fizzled 5 expression

Supplementary Materials

Supplementary Table S1: The potential sequences in the 3′UTR of Frizzled 4 and Frizzled 5 for miR-224 binding

	Predicted sequences (top) for miR-1 (bottom) binding
Position 863-869 of FZD4 3′ UTR	5′... UGAGAUUUACUAAGUUGACUUAC...
miR-224	3′ UUGCCUUGGUGAUCACUGAAC
Position 3820-3826 of FZD5 3′ UTR	5′... UAUAUGAGAGAUUUGGUGACUUU...
miR-224	3′ UUGCCUUGGUGAUCACUGAAC
